# Bilateral Renal Angiomyolipomas with Invasion of the Renal Vein: A Case Report

**DOI:** 10.1100/tsw.2008.29

**Published:** 2008-02-06

**Authors:** C. Blick, N. Ravindranath, A. Muneer, A. Jones

**Affiliations:** Harold Hopkins Department of Urology, Royal Berkshire Hospital, Reading, UK

**Keywords:** Bilateral angiomyolipoma, partial nephrectomy, renal vein invasion

## Abstract

An angiomyolipoma (AML) is usually a benign, rare, and, more commonly, a unilateral renal tumour. Bilateral tumours are very rare, particularly in the absence of tuberous sclerosis complex. Only in a few isolated cases have features of malignancy been associated with an AML. We present a unique case of bilateral AMLs mimicking invasive tumours in the absence of any other features of tuberous sclerosis complex.

## CASE REPORT

A 64-year-old woman underwent a right hemicolectomy for a pT4, N0 Duke's B adenocarcinoma of the ascending colon. As part of disease staging, a CT of her abdomen was performed, incidentally detecting bilateral renal abnormalities (Fig. 1). The right kidney contained a tumour, measuring 7.5 × 4.5 cm, showing fatty attenuation extending along the renal vein to the inferior vena cava. Radiologically, the lesion resembled an angiomyolipoma (AML); however, the presence of venous extension cast some doubt on the diagnosis. As a result, a right open radical nephrectomy and thrombectomy (a mobile thrombus was discovered in the renal vein) was performed via a rooftop incision. Histology of this right-sided tumour confirmed a HMB45-positive AML invading the renal vein stage pT3b. There was also extension of the tumour into the perirenal fat and there were positive resection margins.

The tumour in the left kidney (Fig. 2) measured 3 cm in diameter and showed reduced attenuation. The lesion was solid and poorly defined from the adjacent renal parenchyma. The radiological findings were consistent with a renal cell carcinoma (RCC). After considering options such as thermal ablation and high-intensity frequency ultrasound (HIFU), an elective left partial nephrectomy was performed, resulting in complete resection of the lesion, which proved to also be an AML.

## DISCUSSION

Although rare, AML is the most common mesenchymal tumour of the kidney. AML is generally considered a benign tumour composed of varying amounts of mature adipose tissue, smooth muscle, and thick-walled blood vessels[1].

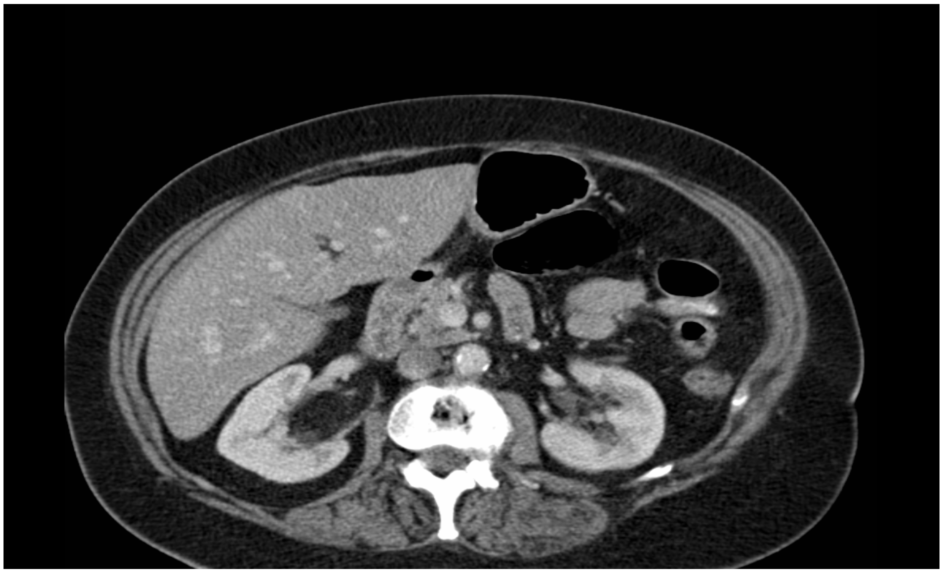

**FIGURE 1**. CT of the abdomen, demonstrating a right-sided tumour invading the renal vein.

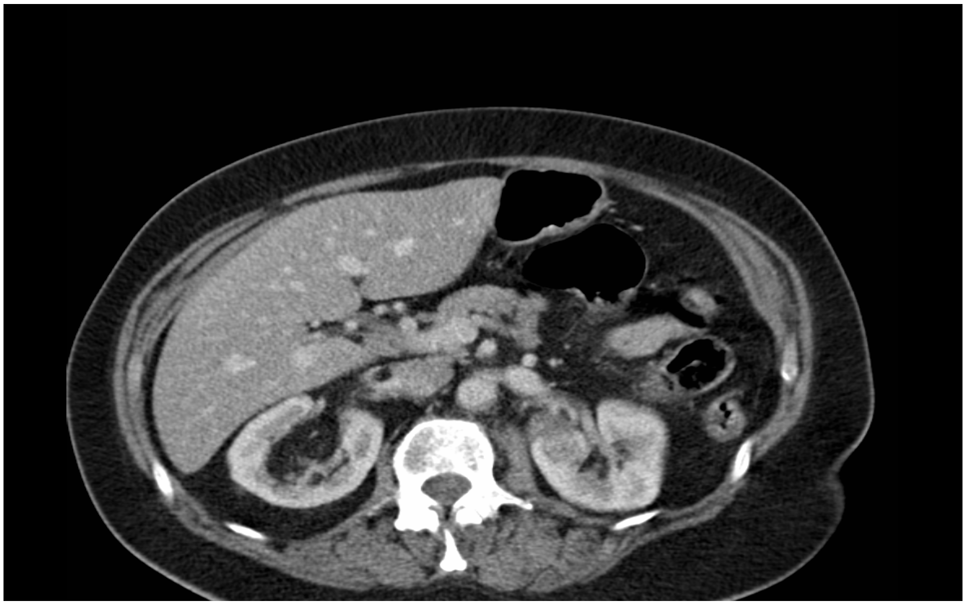

**FIGURE 2**. CT of a soft tissue mass seen in the left kidney.

In surgical series, 50% are sporadic and 50% are associated with tuberous sclerosis, which is a genetic disorder affecting cellular differentiation and proliferation that results in hamartoma formation in many organs[2]. The sporadic forms tend to be large and unilateral. Those associated with tuberous sclerosis are usually bilateral, multiple, and small.

AML almost always follows a benign course, however, extension into surrounding tissues, microscopic changes defining malignancy (such as cellular pleomorphism(3,4,5,6]), hyperchromatic nuclei, and increased mitotic rate have been documented. There have been exceptional cases where the lesion has been lethal, metastasis has been described, and sarcomas have been known to arise from an AML. Current belief is that p53 mutation is associated with malignant transformation in AML.

Diagnosis of AML is often based upon radiological features; numerous fat-fat interfaces produce an intensely echogenic lesion. CT scans can detect fat densities (–70 to –30 Hounsfield units) and the presence of fat is essentially pathognomonic of AML. Histologically, differentiation can be established between AML and RCC by the presence of immunoreactivity for HMB-45. (Figs. 3 and 4)

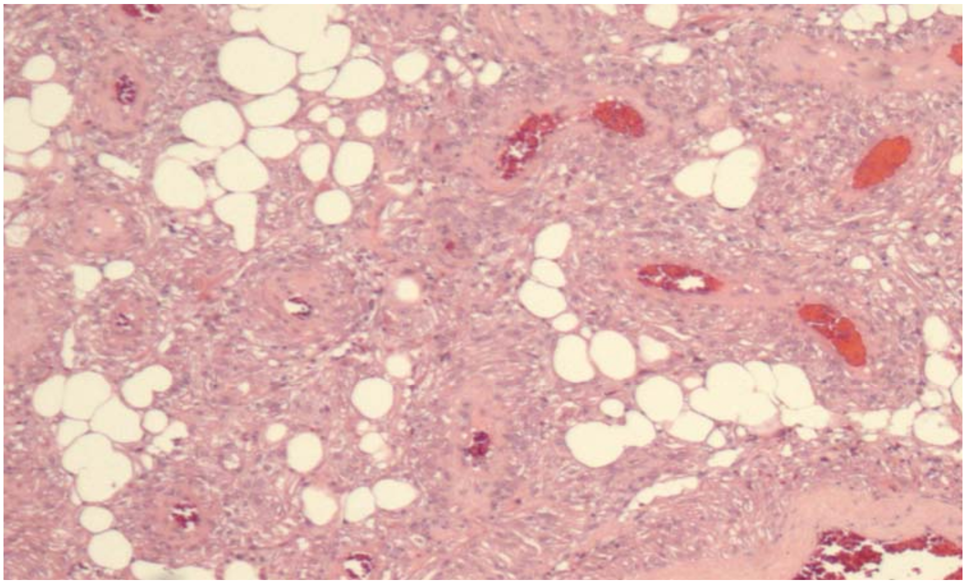

**FIGURE 3.** Angiomyolipoma

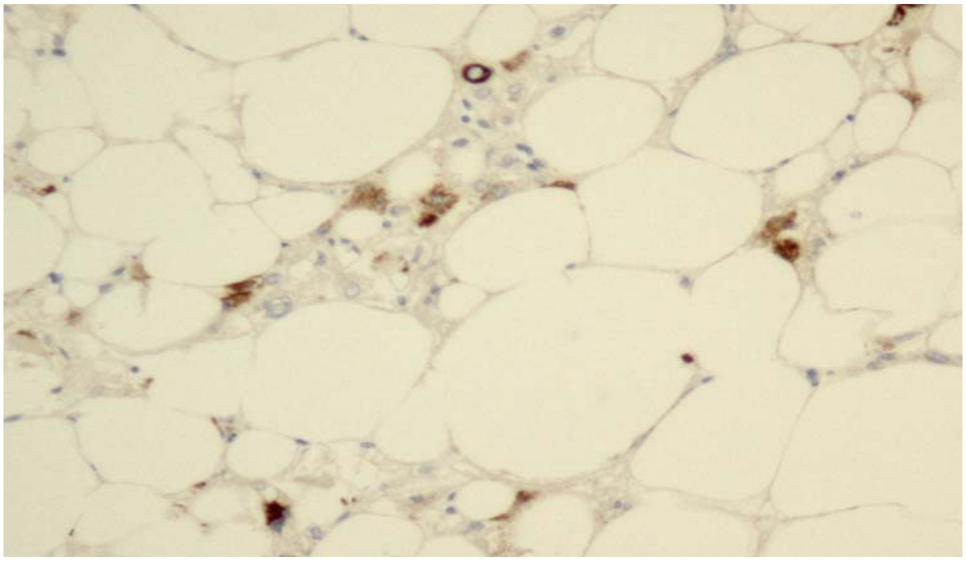

**FIGURE 4.** HMB-45 immunoreactivity

The gold standard management of renal AMLs has not been agreed due to uncertainty over their natural history. In the majority of cases, small lesions of less than 4 cm in diameter are managed conservatively, lesions greater than 4 cm should be monitored closely, and over 50% of patients will require either embolisation, nephron-sparing surgery, or nephrectomy[7]. The literature would suggest that lesions larger than 8 cm require elective intervention either by embolisation, partial nephrectomy, or nephrectomy.

This case report describes a patient with bilateral lesions and some malignant characteristics. The 3-cm lesion would normally have been monitored conservatively, however, due to an uncertainty regarding the benign nature of the lesion, a partial nephrectomy was performed.

Involvement of the renal vein and presence of malignant characteristics in AMLs are rare, and a literature search found 27 previous case reports between 1961 and the present day: 22 were female patients ranging in age from 16–75 years old and four cases were associated with tuberous sclerosis. The most common presentation was flank pain, however, a quarter of the patients were diagnosed as a result of incidental findings[1,2,3,4,5,6,7,8].

This is only the second case of this type where bilateral AMLs have expressed malignant characteristics. The reason behind the behaviour of these tumours is unknown; p53 mutation may well be a causative factor. The fragility of the blood vessel may facilitate aneurysm formation and, hence, intravenous extension. The smooth muscle cells found in tumours with intravenous spread lacked specific proteins, such as calponin h1 and h caldesmon, which are considered to be late differentiation markers of smooth muscle cells. The absence of these proteins may lead to accelerated growth and develop the propensity to spread.

An AML may mimic RCC, however, this is rare and there is insufficient evidence to formulate a recommended management plan to allow for this eventuality. In this case, bilateral renal surgery successfully removed both tumours and the patient avoided dialysis.

## References

1. Cook, J.A., Oliver, K., Mueller, R.F., and Sampson, J. (1996) A cross sectional study of renal involvement in tuberous sclerosis. *J. Med. Genet*. **33,** 480–484.
2. Price, E.G. and Mostofi, F.K. (1965) Symptomatic angiomyolipoma of the kidney. *Cancer* **18,** 761–774.
3. Dodds, L.J., Mishriki, S.F., and Miller, S. (2000) Peripheral renal angiomyolipoma with extension into the renal vein. *Aust. N. Z. J. Surg*. **70(1),** 78–79.
4. Wilson, S.S., Clark, P.E., and Stein, J.P. (2002) Angiomyolipoma with vena cava extension. *Urology* **60(4),** 695–696.
5. Schips, L.,Ratschek, M., Galle, G., Zigeuner, R., Trummer, H., Petritsch, P.H., and Hubmer, G. (2003) Angiomyolipoma with a caval thrombus. *Urol. Int*. **70(4),** 332–334.
6. Kawaguchi, K. et al. (2002) Malignant transformation of renal angiomyolipoma: a case report. *Am. J. Surg. Pathol*. **26(4),** 523–529.
7. Heinke, T., Bonfig, R., and Riedmiller, H. (2002) Nephron-sparing surgery in multiple bilateral angiomyolipomas. *World J. Urol*. **20(4),** 224–226.
8. Eble, J.N. (1998) Angiomyolipoma of kidney. *Semin. Diagn. Pathol*. **15,** 21–40.
